# Excreta flow mapping along the sanitation service chain, a case of Kombolcha town, Ethiopia

**DOI:** 10.1038/s41598-024-53724-7

**Published:** 2024-02-14

**Authors:** Seid Endris, Andinet Kebede, Eshetu Assefa, Almayehu Ali, Tewodrose Desale

**Affiliations:** 1 Kombolcha Institute of Technology, Wollo University, P. O. Box 208, Kombolcha, Ethiopia; 2 Adama Science and Technology University (ASTU), P.O.Box 1888, Adama, Ethiopia; 3 Bahr Dar Institute of Technology, Bahr Dar University, P.O.Box: 26, Bahr Dar, Ethiopia

**Keywords:** Fecal sludge management, Onsite sanitation, Risk exposure, Sanitation service chain, Shit flow diagram, Biochemistry, Biogeochemistry, Environmental sciences, Hydrology

## Abstract

Poor management of fecal sludge (FSM) presents significant risks to public health and the environment. This study employed qualitative and quantitative data collection methods, along with the Shit Flow Diagram (SFD) data analyzing tool to investigate FSM patterns in Kombolcha town, Ethiopia. The findings indicate that 75.7% of housing unites in the town are shared toilets, with multiple households sharing a single facility. The primary toilet technologies used include cistern flush toilets (2.1%), pour/manual flush toilets (19.8%), ventilated improved pit latrines (11.1%), pit latrines with slabs (56.4%), and pit latrines without slabs (10.6%). However, 98.5% of these toilet types had either unlined or only partially lined containments. Furthermore, only 37% of households practice safe pit or sludge tank emptying. As a result, only 17% of fecal sludge goes through the sanitation value chain and is effectively treated, while 39% remains onsite and unemptied, and the remaining 44% is disposed of in a manner that poses risks to the environment and public health. The study highlights the significant public health and environmental risks associated with the high reliance on shared toilets, the prevalence of inadequately lined toilet types, and the low adoption of proper fecal sludge management practices. Addressing these challenges requires the implementation of sanitation bylaws and building code regulations that prioritize hygienic standards and promote improved toilet technologies.

## Introduction

Worldwide, 1.1 billion people practice open defecation and many more do not have services that prevent fecal waste from contaminating the environment. About 2.3 billion people, who still required a basic sanitation service, either use unimproved services or practice open defecation^1^. Similarly, millions use limited sanitation facilities that are shared or communal to households^1^. In urban areas, approximately one billion onsite sanitation facilities are in use worldwide.^2^. However, the typical on-site management system is the accumulation of feces in heavy slime^3^. Fecal sludge without proper management is normally allowed to accumulate in improperly designed pites and drainage canals or dumped into waterways, and resulting in extensive environmental and public health risks^4^.

A lack of safe sanitation systems leads to infection and disease, including diarrhea^4^. Leading causes of disease and death in children under five years in middle and low-income countries^5^ are neglected tropical diseases such as soil-transmitted helminth infections, schistosomiasis, and trachoma that cause a significant burden globally^6^; and vector-borne diseases such as West Nile Virus, lymphatic filariasis and Japanese Encephalitis through poor sanitation facilitating the spread of Culex mosquitos^7^. One in three households in Ethiopia has no toilet facility, leading to open defecation in the bush or fields 39% in rural areas and 7% in urban areas^8^. As a result, diarrhea contributes to more than one in every ten child deaths in the country^9^ . The situation in Kombolcha town is not different. It has been observed that no field research or evaluation has been conducted on the entire fecal sludge management system in Kombolcha town. Moreover, there is a lack of published documentation on comprehensive assessments comprising containment, emptying, transport or conveyance, treatment, and reuse or disposal, based on actual practices. The only available estimations are from WHO and UNICEF at the country level.

As a result, research on fecal sludge management services is crucial for saving lives and safeguarding community health. This study aims to evaluate the management of fecal sludge along the sanitation service chain, identify any gaps in the management process, and isolate the key building blocks for taking action. At present, overflow and leakage of pits, illegal pit/ tank outlet connections to drainage canals, and water bodies are the main problems^10^. These, together with other unsanitary circumstances such as open defecation, lead to extremely serious environmental and community health hazards. To overcome the fecal management limitations in the study area, research on entire fecal sludge management gaps along the sanitation delivery chain from containment up to end disposal/reuse is of paramount importance. Thus, this research was conducted to map excreta flows from containment up to end disposal to show the management gaps at each stage of the service chain and to provide baseline data for future intervention planning.

## Methods

### Study area

**Figure 1:**
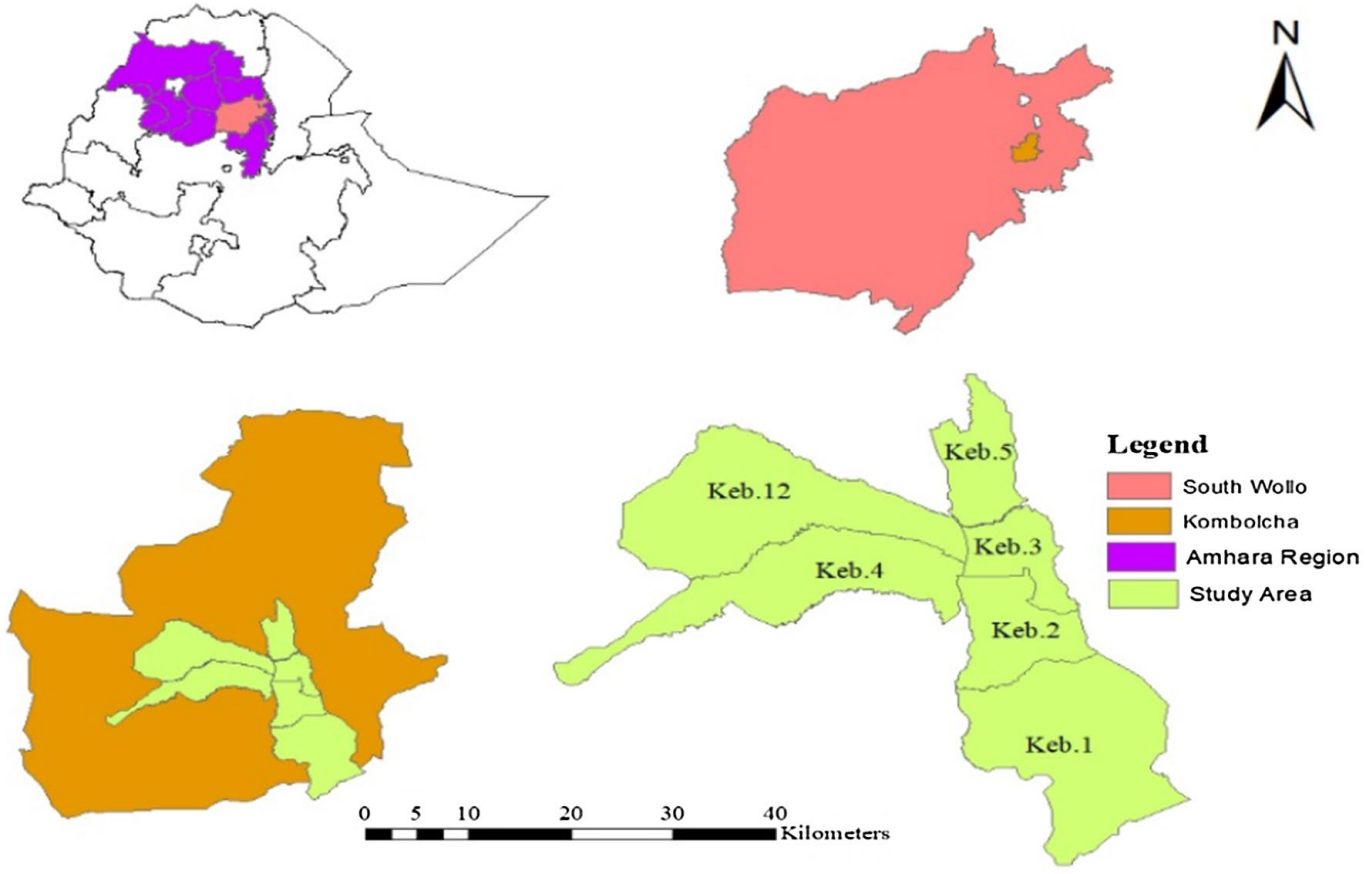
Location map of Kombolcha town.

The study was conducted in Kombolcha town which is located in South Wallo Zone of the Amhara Region, north-central part of Ethiopia (Fig. 1). The town is about 377 km north of Addis Ababa (capital city of Ethiopia) and 505km from Bahr Dar City, the Amhara region capital. Kombolcha town consists of six administrative districts or “kebeles” as shown in green in Fig. 1, with a population estimated at 110,654 and a total of 22,984 households^11^. The altitude ranges from 1842 to 1915 m above sea level. The mean annual rainfall varies from 725.1 to 1361.6 mm and the mean annual temperature varies from 18.7 to 20.9 °C^12^.

The housing types of the town can be classified into government rental houses, townhouses (narrow homes that share walls and toilets with neighboring units), private homes and comdominium housing (a building with a maximum of four floors having multi housing unites ranges from 8 to 10 per floor that has a single toilet per housing unit). Approximately 379 government rental houses are occupied by low-income households. The remaining housing stock consists of around 7722 private houses, 931 townhouses, and 1545 condominium housing units according to the Kombolcha Town administration in 2019.

Currently, there is no available data regarding the distribution of shared or private toilet facilities by housing type and by flush, VIP, and pit latrine technologies in Kombolcha town?. The only existing data is from the 2007 Centeral Statistical Authority (CSA) housing unit census, which provides information on the types of toilet facilities for 15,261 households in the town^13^. Of these households, 3,505 (23%) had no toilet facility, while 861 (5.6%), 1,760 (11.6%), and 9,129 (59.8%) had access to a flush toilet, VIP latrine, and pit latrine technology, respectively, which may or may not have been shared.

### Research design

Fecal sludge in an onsite sanitation containment system can be categorized into fecal sludge (FS) that either stays within or escaped the waste containment structure. Fecal sludge is considered as not contained when it percolates into the ground and contaminates a high groundwater table or if it flows out of the tank or pit through an open-drain/water body/open ground^14^.

The research design is primarily based on the Shit Flow Diagram (SFD) tool administered by the Shit Flow Diagram Promotion Initiative (SFD-PI) which is found under the set of diagnostic tools developed by the World Bank^15^. Thus, the framework for data collection was based on the SFD methodology framework for FSM diagnostics. This SFD methodology was selected because the study focused on fecal sludge management and SFD is currently advocated worldwide as a systematic fecal sludge management assessment tool. The SFD tool can be used to understand the current FS management patterns.

### Data collection

The study used two quantitative (i.e. a housing unit survey and field observations) and three qualitative (i.e. key informant interviews (KII), focus group discussions (FGD) and literature review) data collection methods. It was important to conduct a literature review to gather relevant data and background for the research to be abundantly informed and complete. Literature was gathered from various sources such as relevant journals, conference proceedings, academic thesis, organizations’ and donor reports, official records or reports and maps from Kombolcha town administrative areas, the Susana website (https://www.susana.org/en/#), key informants and sanitation professionals working in Kombolcha town.

To evaluate the types of toilets and fecal sludge management (FSM) arrangements within housing units, a housing unit survey was conducted. The survey consisted of 58 questions, where 25% of the answers were collected through direct observation by enumerators. Within each sampled housing unit, the house owner was interveiwed, or where no owner was present, a tenant household was interviewed. For the interviews with key informant stakeholders in the sanitation sector, checklists were established from which interviews were conducted to bring to light the present state of management practices. Key informants included vacuumed truck drivers, the water supply and sewerage service office, Kombolcha town municipal authority, Kombolcha town health department, sanitation and beautification department, Kombolcha town health extension workers, the faecal sludge treatment plant (FSTP) supervisor and attendants, flush toilet and pit latrine installers/masons, and public toilet attendants.

Visual inspection and observations were embarked along each stage of the sanitation delivery/service chain, from toilet containment up to treatment or disposal of fecal sludge during transect walks which were conducted in four of the six districts/kebeles of the study area. Informal interviews during transect walks were also carried out using a checklist.

These were systematic walks aimed at observing the manner in which sludge pit emptiers discharged sludge at disposal sites and checking the frequency at which they went there, methods used for end-use/disposal of FS, location of water supply source from disposal sites, access for emptying of pits and tanks, risk of fecal sludge exposures along the service chain, and the distribution of sanitation system technologies across the town.

Besides these, two focus group discussions (FGD) were held with community representatives as well as slum dewellers and conducted at the end of the field-based research. The sessions were limited to two because of resources constraints. The quantitative data collected through the housing unit survey concerning containment technologies was adjusted, regarding illegal toilet outlet connections that could not be observed during the survey, and rates of open defecation. The FGD topics also focused on obtaining data related to household practices, service levels, past interventions, risks, and other issues associated with fecal sludge management services. Those topics were based on fecal sludge management global study data collection instruments^16^.

### Sampling approach

A stratified sampling method was used to select a sample of residential housing units for the quantitative survey. The list of forty (40) wards of the 6 urban districts/kebeles of Kombolcha town (Fig. 1) with their respective population and number of housing unit was collected. In cases where adjacent wards were found to be similar in terms of population density, land use composition, and built-form, they were merged to generate a cluster, after reconnaissance survey. Thus, nine (9) clusters were identified for this study. Next, the sample size was determined by considering financial constraints, time, the purpose of the study, and representativeness.

The sample size was estimated using the expression given by^17^:

$$ n = \frac{{\left[ {Np\left( {1 - p} \right)} \right]}}{{[(e^{2} /Z^{2} *\left( {N - 1} \right) + p*\left( {1 - p} \right)]}} $$

where n = the required sample size; N = Total number of housing units; Z = standard normal deviation; e = sampling error; P = the proportion of targeted population with having toilet facilities 80%. The number of households (22,984) was obtained from the Kombolcha town administration council. Therefore, with a 95% confidence level, ± 5% precision the minimum sample size of housing unit was n = 243.

Finally, a sample of 243 was distributed across the 9 clusters proportional to their population size, with 17, 18, 37, 22, 12, 50, 51, 27, and 9 households, each in a separate housing unit, representing each cluster. These sample housing unites were drawn for data collection using a simple random sampling method since its assumed to have similar characteristics. Thus each sampled household is assumed to represent the specific onsite sanitation technology and practices of residents of that housing unit. If multiple households shared a toilet, only one representative household was chosen for sampling. To ensure validity, the questionnaires were translated into the local language (Amharic), and pilot tests were conducted.

On the other hand, in a qualitative study, purposive sampling was conducted for stakeholder identification and key informant interview process. Appropriate checklists were established and 24 key informant interviews (KIIs) were conducted with different stakeholders from the government and non-government organizations.

### Data analysis and ethical considerations

Data analysis was performed in two stages. First, a combination of quantitative and qualitative data was pre-analyzed, that is the collected quantitative data were analyzed using Statistical Package for Social Science (SPSS) and Micro Soft Excel for the preparation of data input. The qualitative data from Key Informants Interview (KII), field observations and FGD were compiled and analyzed through the description, narrating and interpreting the situation contextually.

Secondly, the survey quantitative data regarding the interviewed household’s information, house ownership, containment technology, emptying information, and the like were analyzed using SPSS and Microsoft Excel. The pre-analyzed quantitative data with Micro Soft Excel and SPSS tools were prepared for further analysis with the Shit Flow Diagram (SFD) analyzing tool. As detailed byScott^15^, SFD is an advocating tool that signifies where the faecal waste ends up, what proportion is safely managed, and where the unsafely managed portion is delivered. Furthermore, SFD provides a convincing visual summary by highlighting at which stages the fecal waste becomes unsafely managed for a given population^18^. Thus, the excreta flow was mapped/analyzed from point of defecation to end-use or disposal using the SFD tool with color representation in which red and green were used to represent unsafely and safely managed excreta, respectively.

For the research ethics, a, written research permit with BiT academic council BiT Senate No 10/2012 on Feb. 27/2020, was granted by Bahr Dar University of Ethiopia. During the residential housing unit’s sanitation technology study, the objective was to inform interviewees of the purpose of the study and obtain their oral consent. Participants were given the freedom to withdraw or halt the interview at their discretion and had the option to select which questions they felt comfortable answering.

### Limitations of the study

To ensure the accuracy and minimize bias, data for this research has been collected from various sources and methods. However, there were gaps in the quantitative data, particularly for the formation of the SFD. As a result, quality issues are discussed below.

Insufficient information from households and limited published surveys made detailed analysis of containment technologies challenging. Most surveys/reports differentiate between user toilet interfaces, rather than providing insights into containment systems below the ground. Estimations on tank and pit latrine containment systems relied on KII with pit/tank installers (masons), and FGD with technical staff from the sanitation department of the municipality to validate and cross-check the survey data.

Furthermore, there was a limited data in differentiating the amount of faecal sludge (FS) generated at a housing unit level which corresponding to the amount FS generated by the interviewed household at that unit. To adjust the data clarity in one- to -one correspondence, the sample size of housing unit was calculated based on the total number of households.Likewise, when questioning stakeholders about the amounts of fecal sludge transported and disposed at the plant and the plant treatment efficiency, there was an information gap or missing data. In addition, open defecation practice estimation was difficult, even though the wide range of its practice in all parts of the city was witnessed. Following this, reasonable/rational estimations and assumptions have been made for credibility and accuracy of the SFD and are discussed below.

## Results and discussion

### House and toilet occupancy

Out of the 243 representative sample housing unites, 69.1% were privately owned houses. The survey did not randomly select respondents within multiple housing units, but instead relied on landlords as the primary source of information regarding fecal sludge management arrangements. In cases (11.9%) where landlords were unavailable, tenants were interviewed. Of the 243 sample housing units surveyed, 81% were occupied by tenants of rental roomwithin the landlord's compound (a mix of tenants and landlords). The rest 19% of sample housing units were government-owned and occupied by tenants or townhouses. Among these, 46% were shared house owners or townhouses, referring to narrow homes that share walls and toilets with neighboring units.

The survey showed that 24.3% of the respondents had access to private household toilets and 75.7% of Kombolcha town inhabitants rely on shared toilets between 2 or more households. This is because of the increase in the construction of rental rooms attached to theandlord’s house with common toilets, following the need for housing in the town. So, out of 75.7% of shared toilet user households, 79.4% shared the toilet with their landlords and other tenants on the property. The remaining 20.6% households utilized communal toilets that were shared among tenants of government-owned houses and/or townhouses. Public toilet user households and households without toilet facilities were neither observed nor reported during the household survey. However, for the purposes of this study to develop the SFD, it was necessary to include open defecation, considered to be 7% as estimated by WHO and UNICEF,^1^ as the country-level figure for Ethiopian urban towns and cities.

### On-site sanitation technologies

**Figure 2:**
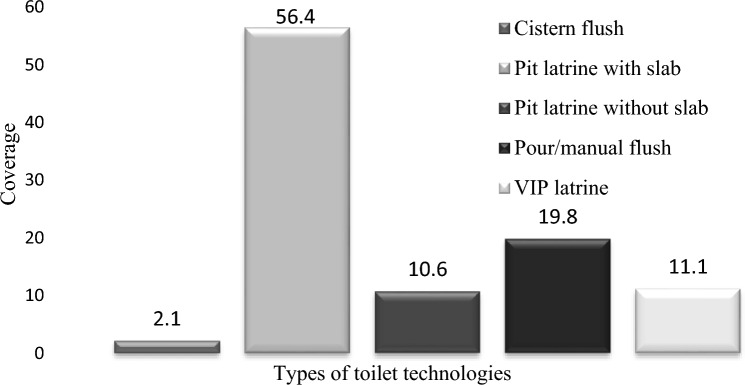
Toilet technologies and their coverage (%).

The survey results showed that there are five main types of household toilet technologies in Kombolcha town; namely, cistern flush toilets, pour/manual flush toilets, Ventilated Improved Pit latrine (VIP), pit latrine with and without slab. An estimated 56.4% of the sampled households residents used a simple pit latrine with a slab; where as, cistern flush toilet, pour/manual flush toilet, VIP latrine, and pit latrine without slab technologies were used by 2.1, 19.8, 11.1 and 10.6%, respectively (Fig. 2).

There was no currently existing data on the Kombolcha town’s toilet technology types except for the 2007 data from Central Statistical Authority^8^ of housing units and their respective types of toilet facility, which showed that of all of Kombolcha town housing units, 23% had no toilet facility; whereas, 5.6, 11.6 and 59.8 of them had flush toilets, VIP latrines, and pit latrine technologies, with unknown sharing status. The present findings differ from the CSA report of conditions in 2007, especially for the no toilet facility and flush toilets categories. However, result of this study is almost the same as that of the CSA report for the housing units with VIP latrine.

More than three-fourths of Kombolcha town inhabitants use pit latrine technologies including VIP latrine. However, flush toilets were significantly low; out of all the housing units surveyed, only 21.9% had flush toilets.This percentage includes 2.1% with cistern flush and 19.8% with pour flush toilets (Fig. 2).

According to WHO and UNICEF^1^ Sustainable Development Goal (SDG), improved sanitation facilities are categorized as safely managed, basic, and limited facilities. Improved facilities that are not shared count as either basic or safely managed services. The estimated study result is presented in this report (Table 1).

**Table 1:** Sanitation facility used, by Joint Monitoring Program (JMP) category.

Type of facilities	Categories	Percentage
	Basic facilities (not shared with other households)	17.3
Improved facilities	Limited facilities (shared between two or more households)	72
Unimproved facilities	Use of pit latrines without a slab	10.7

cOn the other hand, open defection is time and again encountered in Kombolcha town and it has been practiced mainly by the homeless, as they do not have sanitation facilities. Discussion with community members together with observations during field survey showed that open defecation is practiced in almost all parts of the town, especially the older districts: either by newcomers arriving from remote areas for a work opportunity or market exchange purpose. Although the current open defecation practice is expected to even get worse, the extent could not be determined due to lack of census data or a reasonable estimated number of people who practice open defecation. For the purposes of this study, it is considered to be 7% which was estimated by WHO & UNICEF^1^ as a country level for Ethiopian urban areas.

The FGD and KII showed that government owend individual rental house communal toilet users or townhouses of slum areas share one seat for 25–30 households whereas households sharing houses typically share one seat for three households. The transect walk in vulnerable areas and observation during the survey showed poor management of the communal toilet facilities, such as infrequent cleaning and desludging of the facility, and poor maintenance practices related to the dense population settlements that make management difficult. Every household is responsible for the operation and maintenance tasks of the communal toilet including emptying and maintenance fees. However,frequent filling of tanks and pits together with long waiting time to get emptying service results in a health risk exposure.

### Risk of Groundwater contamination

Due to the lack of available groundwater maps or data concerning the actual groundwater levels of the town, the estimations of groundwater polluation were made based on HH survey, literature review, and key informant interviews. The risk of groundwater pollution was calculated using the SFD graphic generator groundwater assessment helper tool. The risk of groundwater pollution was estimated from data on drinking water from private groundwater sources, vulnerability of the aquifer, and the distance between groundwater sources and sanitation facilities. Among the 243 sampled housing units, 3.7% of them had a private well for non-drinking purposes as drinking tap water is available from the municipality, and were identified as a low risk of groundwater pollution. However, from the sampled 243 housing unites 3.2% of the population live in areas with a significant risk of groundwater pollution.

### Containment system estimations

Analysis of the KIIs, FGD, survey, and visual inspections during the survey led to the following quantitative estimations for onsite sanitation technologies and their respective containment systems as shown in Table 2, except for the 7% country-level open defecation practice based on WHO and UNICEF^1^ estimation. Those estimations were made taking into account the survey underestimation of illegal practices that household’s were reluctant to admit. Thus, more weight was given to KII statements, especially for illegal toilet outlet connections and related issues. However, the estimations were challenging, as a lot of different systems exist in the town that differ from SFD system terminology for both tanks and pits. As a result, there was a need for grouping of similar technologies into SFD system terminology categories, for both tank and pit latrine systems (Table 2).

**Table 2:** Final estimations for the SFD matrix containment calculations.

Containment technologies	Estimations (%)
Fully lined tank/pit, no outlet or overflow	1.5
Lined tank/pit with impermeable walls and open bottom, no outlet	10.0
Lined/partially lined tank/pit discharged to open drain/water body	9.5
Lined pit/tank with semi-permeable walls and open bottom, no outlet	46.0
Unlined pit with no outlet or overflow	11.0
Pit latrines abandoned when full and covered with soil, no outlet	8.0
Pit latrines abandoned when full and covered with soil, no outlet or overflow in significant GW risk areas	3.2
Containment failed, damaged, collapsed, or flooded—no overflow	3.8
Open defecation	7.0

The study findings show that fecal waste collected at 98.5% of the on-site sanitation facilities was discharged into partially lined or compeletly unlined containment systems . Out of the sampled 243 housing units, 8% and 3.8% were safely abandoned and damaged/collapsed pits, respectively. And 3.2% of containments were likely the major causes of groundwater pollution through which the faecal sludge infiltrates into the ground (Table 2).

### Faecal sludge emptying

The municipality has one vacuum truck of 8 m^3^ capacity and the governmental university in the town, Wollo University, also has one vacuum truck, which provids service for the University only. The municipality can not provide sufficient emptying services with the single truck and there no private trucks in the town to provide emptying services. Instead, private vacuum trucks with a capacity ranging from 6 to 10 m^3^ come on request from a nearby town called Dessie, which is located about 23 km from Kombolcha. Based on the emptying service provider's interview and survey results, it costs approximately $ 13 per trip to get the emptying services with the municipal vacuum truck, whereas the private emptying service charge varies from $ 31 to 57. Private vacuum truck operators pay $ 5 per truck to empty their contents at the fecal sludge treatment plant.

The study found that 37% of households with an emptiable toilet had experienced a pit/tank filling up. They emptied their toilet facility and reused it again. Among households that had emptied, 5.8% (2.1% of all households) reported pit overflow occurring due to lack of emptying service when needed, 51% reported that their pit/ tank emptying frequency was less than one year while 12.2% reported a frequency of 4 and above years (8.9% in 4 to 6 years and 3.3% above 6 years). The remaining 63% of pits or tanks were determined as emptiable facilities that have never been emptied before, technologies that were unable to be empty, and emptiable technologies with illegal outlet connections. In total, 78% (100% − (7 + 3.8 + 3.2 + 8)) of onsite facilities were emptiable (except open defecation, damaged/collapse, and fully abandoned pits) (Table 2) . Out of 78% of emptiable toilet facilities, 41% (78–37%) were not emptied before. Out of 41% of emptiable but unemptied technologies, 9.5% illegally connect their outlet into an open drain/water body. The remain 31.5% is contained and emptiable but not yet emptied. In addition, the contained and unemptiable toile facilities are 8% which are safely abandoned pits. Thus, the contained and not emptied toilets are 39.5% (31.5% + 8%)Table 2.

### Faecal sludge transport

There was limited evidence of vacuum trucks dumping FS to land or parts of the town environment before reaching the treatment plant and fecal sludge being removed informally by households themselves. Also, it was observed that the FSTP was located only at 3 to 3.5 km from the center of the town, and its access road was suitable for haulage of FS during rainy seasons. Similarly, the municipality has a measure restricting those discharging the FS into the environment instead of FSTP. Thus, by taking into consideration the above enabling situations, all of FS removed from pits and tanks was considered as delivered to the fecal sludge drying beds but 90% was applied (see Table 3, last column) rather than assuming 100% because there are possibilities which the vacuumed unload the faecal sludge out the drying bed cells.

**Table 3:** Estimations on FS emptying of on-site sanitation systems/technologies.

Onsite sanitation technologies	% emptaible containments	Emptied faecal sludge (%)	faecal sludge delivered to FSTP (90%)
Fully lined tank, no outlet	1.5	1.50	1.30
Lined tank with impermeable walls and open bottom, no outlet	10.0	6.86	6.20
Lined pit/tank with semi-permeable walls and open bottom, no outlet or overflow	46.0	25.90	23.40
Unlined pit with no outlet	11.0	2.75	2.50
Total emptied FS		37.10	33.40

### Faecal sludge treatment

Currently, Kombolcha has a fecal sludge treatment plant (FSTP) with four main components; unplanted drying beds, maturation ponds, storage lagoons, and sanitary landfills. The plant is located in the lowland area, at about 3.5 km away from the town center and uphill of the Borkena River about 70 m away. According to Kombolcha town water supply and sewerage service authority^19^, a total of 12,733, 17,822 and 21,602 m^3^ of fecal sludge have been delivered to the plant from Kombolcha and its neighboring towns, for the years 2017, 2018, and 2019, respectively.

Poor and damaged retaining and common walls, plant growth on the drying beds at rest, non-functional screening unit, and loss of periodic cake or dry fecal sludge removal are the current problematic situation of the treatment system. Furthermore, the vacuum trucks empty sludge onto the drying bed with dried sludge/cake. This situation results in accumulation of rubble and trash, which clog pore spaces within the sludge and prevent draining the liquid, resulting in rewetting of the sludge and prolonged the drying period.

The efficiency of treatment at the fecal sludge drying beds was not determined but the plant is not operating optimally. Thus, a figure of 50% treatment efficiency was used to produce SFD. This estimation was drawn based on SFD-PI^14^ methodology for unknown data on the efficiency of the plant, limited evidence on its operation, and self-judgments after repeated field visits as well as referring to previous SFD preparations^20^.

**Figure 3:**
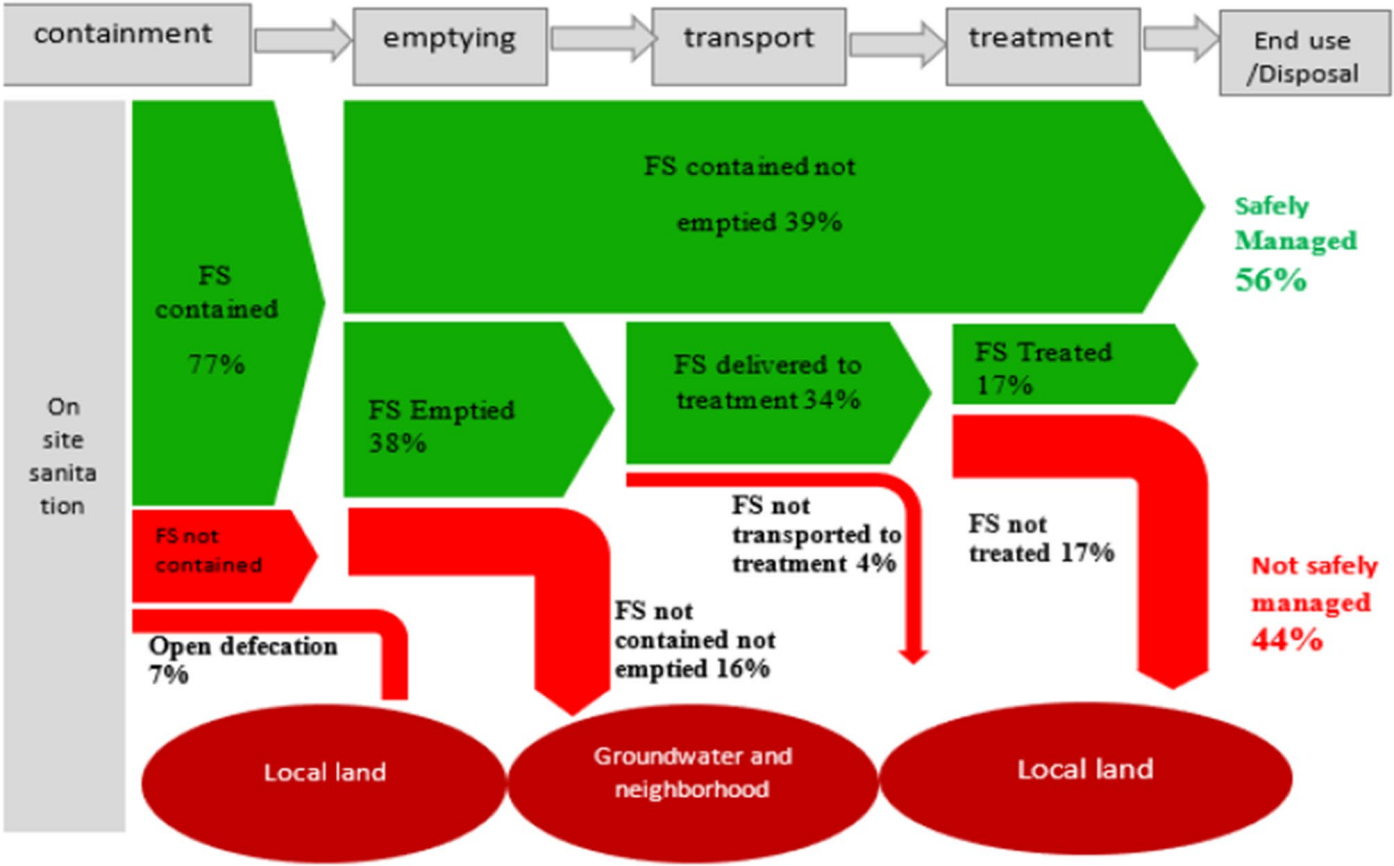
Citywide excreta flow diagram.

The study presents the overall FS emptying of on-site sanitation technologies. The percentage values of emptying for each containment technologies were determined from containment types and their respective emptying practices (Table 3). The evacuated containment technologies, their emptying practices and 50% treatment efficiency together with the percentage of the population using each type of on-site sanitation system were employed to develop the shit flow diagram as the SFD considers percentages of population rather than housing units (Fig. 3).

As indicated in the SFD, there is 93% coverage of on-site sanitation and 7% open defecation. The excreta flow diagram highlights that 77% of fecal sludge is contained onsite (emptaible and unemptaible containments which is 68.5% and 8% respectively). Out the total faecal sludge 39% is unemptied and considered as contained onsite. This can be considered as safely managed at present. However, the situation is intended for change over time. Besides, there is an increase in levels of groundwater contamination risk due to increased reliance on poorly constructed unlined pits, resulting in soil saturation and groundwater table contamination. All these situations will contribute to a greater quantity of the unmanageable fecal sludge, with possibly increasing risk to the environment and public health. The study also found out 16% uncontained on-site sanitation technologies, in which FS infiltrates into the ground and pollutes the groundwater or FS from the technologies flow through open-drain/water body/open ground and potentially cause serious health and environmental hazards (Fig. 3).

What is clear from the SFD is also that almost half, about 56%, of fecal sludge in the town is safely managed. From the contained FS, only 17% of it passes through the sanitation service chain and is safely managed, while the rest (39% of all fecal sludge produced) remains safely contained and unemptied without risk to public health or environmental contamination through groundwater pollution or direct exposure. However, it can still have negative impacts on the environment and the health of the community as waste in the toilet can continue to decompose and release harmful gases like methane and hydrogen sulfide, which can contribute to air pollution and respiratory health problems. The management of fecal sludge deteriorates when there is limited space to dig new pits instead of emptying the existing ones. Likewise, 44% faecal sludge, which is the unsafely managed portion, poses environmental and health risk exposures directly or by contaminating groundwater. It is also shown that the households with a safe containment facility that has been emptied at least once are only less than half of all households (38%). Of these, 90% of the emptied fecal sludge is estimated to be delivered to the treatment plant (FSTP), with an estimated 50% treatment efficiency, resulting only 17% of all fecal sludge in Kombucha town being effectively treated (Fig. 3).

### Implications of the study and potential areas for future research

The high percentage of households relying on shared toilets and the dominant use of pit toilets with and without slabs are not hygienic and require the enforcement of sanitation bylaws and building code regulations. The unsafely managed portion of the fecal sludge is posing environmental and health risk exposures directly or by contaminating the groundwater sources. The study suggests identifying priority areas for the introduction of a range of sewerage options and the need to consider the use of well-designed septic tanks, anaerobic baffled reactors, or other amended on-site containment systems supported by more responsive fecal sludge management services.

Future research areas could include exploring the feasibility of implementing the recommended sewerage options and containment systems in Kombolcha town. Additionally, research could focus on identifying the most effective ways to enforce sanitation bylaws and building code regulations to ensure the quality of service and promote hygienic conditions.

## Conclusions

In conclusion, 75.7% of households in Kombolcha town rely on shared toilets, and the dominant toilet technology is a pit toilet with and without a slab, which account for 67.1% of all facilities. This is an unsustainable way of fecal sludge management that deteriorates the environment and public health unless sanitation bylaws and building code regulations are enforced. Standards of latrine construction and management must be set to address the required quality of service and to promote hygienic conditions.

In the containment and emptying stage, most on-site sanitation facilities were only partially lined or completely unlined. The quality of construction and surrounding soil conditions greatly influence fecal sludge management within the containment facility, resulting in tanks and pits functioning for several years without the need for evacuation. However, as the population density increases due to the introduction of new industries, this practice will become increasingly unsustainable as the soil's absorption capacity becomes exceeded.

Furthermore, the developed Shit Flow Diagram (SFD) indicated that 56% of fecal sludge is safely managed at the moment, most of it due to remaining unemptied so far. A total of only 17% of all fecal sludge currently passes through the each stage of sanitation service chain safely and gets effectively treated. The remaining 44% of fecal sludge is unsafely managed i.e., poses environmental and health risk exposures directly or by contaminating the groundwater sources. Insights from this study can inform priority areas for the introduction of a range of sewerage options and the need to consider the use of well-designed septic tanks, anaerobic baffled reactors, or other amended on-site containment systems supported by more responsive fecal sludge management services.

## Data Availability

All data generated and analyzed during this study are included in this published article.
